# The role of Arabidopsis Actin-Related Protein 3 in amyloplast sedimentation and polar auxin transport in root gravitropism

**DOI:** 10.1093/jxb/erw294

**Published:** 2016-07-29

**Authors:** Jun-Jie Zou, Zhong-Yu Zheng, Shan Xue, Han-Hai Li, Yu-Ren Wang, Jie Le

**Affiliations:** ^1^Key Laboratory of Plant Molecular Physiology, CAS Center for Excellence in Molecular Plant Sciences, Institute of Botany, Chinese Academy of Sciences, Beijing 100093, China; ^2^Key Laboratory of Microgravity, Institute of Mechanics, Chinese Academy of Sciences, Beijing 100190, China; ^3^University of Chinese Academy of Sciences, 19A Yuquan Road, Beijing 100049, China

**Keywords:** Amyloplast, arabidopsis, ARP2/3, auxin, gravitropism, PINs.

## Abstract

Gravitropism is vital for shaping directional plant growth in response to the forces of gravity. Signals perceived in the gravity-sensing cells can be converted into biochemical signals and transmitted. Sedimentation of amyloplasts in the columella cells triggers asymmetric auxin redistribution in root tips, leading to downward root growth. The actin cytoskeleton is thought to play an important role in root gravitropism, although the molecular mechanism has not been resolved. *DISTORTED1* (*DIS1*) encodes the ARP3 subunit of the Arabidopsis Actin-Related Protein 2/3 (ARP2/3) complex, and the *ARP3/DIS1* mutant *dis1-1* showed delayed root curvature after gravity stimulation. Microrheological analysis revealed that the high apparent viscosity within *dis1-1* central columella cells is closely associated with abnormal movement trajectories of amyloplasts. Analysis using a sensitive auxin input reporter DII-VENUS showed that asymmetric auxin redistribution was reduced in the root tips of *dis1-1*, and the actin-disrupting drug Latrunculin B increased the asymmetric auxin redistribution. An uptake assay using the membrane-selective dye FM4-64 indicated that endocytosis was decelerated in *dis1-1* root epidermal cells. Treatment and wash-out with Brefeldin A, which inhibits protein transport from the endoplasmic reticulum to the Golgi apparatus, showed that cycling of the auxin-transporter PIN-FORMED (PIN) proteins to the plasma membrane was also suppressed in *dis1-1* roots. The results reveal that ARP3/DIS1 acts in root gravitropism by affecting amyloplast sedimentation and PIN-mediated polar auxin transport through regulation of PIN protein trafficking.

## Highlight

Arabidopsis actin-related protein ARP3 plays a role in amyloplast sedimentation and polar auxin redistribution during root gravitropism.

## Introduction

Plants can sense changes in their postion relative to the gravity vector and use this to redirect the growth of their organs for optimal growth and development. Plant gravitropism consists of three major phases: gravity sensing, gravity signal transduction, and gravitropic response (Blancaflor and Masson, 2003). In higher plants, the sensing site in roots is believed to be in the columella cells of the root caps, whereas in shoots gravity sensing occurs in the endodermal cells (Morita, 2010). Amyloplast sedimentation/movement in the gravity-sensing cells is important for gravity perception. According to the starch-statolith hypothesis, the sedimentation of amyloplasts (statoliths) in gravity-sensing cells (statocytes) can trigger the conversion of gravitational potential energy into biochemical signals (Kiss, 2000; Leitz *et al.*, 2009; Hashiguchi *et al.*, 2013; Toyota *et al.*, 2013). The Cholodny–Went theory proposed that asymmetric auxin redistribution between the upper and lower side of root or shoot triggers differential growth, resulting in the downward growth of roots and upright growth of shoots. Additional studies have supported the notion that asymmetric auxin redistribution is important for root gravitropism (Swarup *et al.*, 2005; Vanneste and Friml, 2009; Band *et al.*, 2012).

Actin filaments not only provide mechanical support for cells but also are involved in a variety of biological events (Pollard, 2007). It has been proposed that the actin cytoskeleton is a major regulator of gravitropism (Blancaflor, 2002); however, studies using actin cytoskeleton inhibitors have produced contradictory results regarding their effects on gravitropism (Yamamoto and Kiss, 2002; Hou *et al.*, 2003, 2004; Palmieri and Kiss, 2005; Mancuso *et al.*, 2006). Recently, genetic studies have begun to uncover the molecular mechanisms behind how the actin cytoskeleton plays a role in gravitropism. In Arabidopsis (*Arabidopsis thaliana*) root tips, the central columella (CC) cells have a finer and less robust network of filamentous actin (F-actin) arrays in contrast to the prominent actin bundles in peripheral columella (PC) or lateral root cap (LRC) cells (Blancaflor, 2013). By contrast, the endodermal cells in inflorescence stems contain a network of distinct F-actin bundles (Saito *et al.*, 2005; Zhang *et al.*, 2011). The non-homogeneous structures in statocytes arising from intracellular components such as cytoskeletons and endomembranes have been shown to significantly affect the complex movements of amyloplasts (Saito *et al.*, 2005; Nakamura *et al.*, 2011). Treatment with the actin-disrupting drug Latrunculin B (Lat B) can increase the sedimentation of amyloplasts in the columella cells and promote root curvature in Arabidopsis (Hou *et al.*, 2004). *ALTERED RESPONSE TO GRAVITY1* (*ARG1*), which encodes a DnaJ-like protein, potentially interacts with the actin cytoskeleton and is required for hypocotyl gravitropism through the regulation of amyloplast movement (Sedbrook *et al.*, 1999; Shiva Kumar *et al.*, 2008). An Arabidopsis E3 ligase SHOOT GRAVITROPISM9 (SGR9) localizes to endodermal amyloplasts and promotes detachment of amyloplasts from actin bundles, allowing the amyloplasts to sedimentate during shoot gravity sensing (Nakamura *et al.*, 2011). These observations demonstrate the role of the actin cytoskeleton in gravitropism by affecting amyloplast movement. Recently, the introduction of microrheological analysis has revealed the relationship between actin organization and amyloplast sedimentation in the columella cells (Zheng *et al.*, 2015).

The role of auxin in linking gravity sensing to response has been well established (Sato *et al.*, 2015). Asymmetric auxin redistribution between the upper and lower sides of gravity-stimulated roots causes differential growth in the root elongation zone, resulting in root curvature. It has been proposed that the actin cytoskeleton also plays an important role in the growth response phase of gravitropism by regulating auxin transport (Blancaflor, 2013). Auxin transport is mainly mediated by auxin transporters, including PIN-FORMED (PIN) group proteins (Vanneste and Friml, 2009). ARG1 and ARG1-LIKE2 (ARL2) are required for PIN3 relocalization and asymmetrical redistribution of auxin upon gravity stimulation (Harrison and Masson, 2008). Arabidopsis SPIKE1 (SPK1), which belongs to the conserved DHR2-Dock family of Rho guanine nucleotide exchange factors (Qiu *et al.*, 2002; Basu *et al.*, 2008), is required for RHO-LIKE GTPASE FROM PLANTS 6 (ROP6) activation and inhibits PIN2 internalization through the stabilization of actin filaments in roots, modulating auxin redistribution during gravitropic responses (Lin *et al.*, 2012). ROP6 and its downstream ROP-INTERACTIVE CRIB MOTIF-CONTAINING PROTEIN1 (RIC1) are required for auxin-mediated root gravitropism through regulating endocytosis and internalization of PIN1 and PIN2 (Chen *et al.*, 2012). These observations reveal the impacts of the actin cytoskeleton and its signaling pathway on auxin transport during gravitropism.

The organization and function of the actin cytoskeleton are regulated by diverse actin-binding proteins, including profilin, actin-depolymerizing factor, formin, and the Actin-Related Protein 2/3 (ARP2/3) complex (Staiger and Blanchoin, 2006). The ARP2/3 complex produces branched filaments both to push forward the leading edge of motile cells and for endocytosis (Pollard and Borisy, 2003). In Arabidopsis, mutations in the ARP2/3 complex usually lead to distorted trichomes and cause epidermal cell adhesion defects (Le *et al.*, 2003; Mathur *et al.*, 2003; El-Assal *et al.*, 2004). Arabidopsis ARP2/3 complex subunits have also been reported to be involved in stomatal movement (Jiang *et al.*, 2012; Li *et al.*, 2013, 2014) and salt stress (Zhao *et al.*, 2013). Moreover, it has been reported that Arabidopsis *ARP3/DISTORTED1* (*DIS1*) and *ARPC2A/DISTORTED2* (*DIS2*) have different roles in gravitropism and phototropism (Reboulet *et al.*, 2010); however, the mechanism by which *DIS1* is involved in gravitropism remains unclear. It has been hypothesized that DIS1 may affect translocation of auxin transporters or other actin-associated proteins involved in gravitropism (Reboulet *et al.*, 2010). Here we report that ARP3/DIS1 takes part both in amyloplast sedimentation by affecting local apparent viscosity in the central columella cells and in asymmetric auxin redistribution across the root tips through the modulation of PIN cycling.

## Materials and methods

### Plant materials and growth conditions

*Arabidopsis thaliana* ecotype Columbia (Col) was used as the wild type in this study. The *dis1-1* and *dis2-1* mutants were kindly provided by Daniel B. Szymanski (Purdue University, West Lafayette, Indiana, USA). *pPIN2::PIN2-GFP*, *pPIN3::PIN3-GFP*, *pPIN7::PIN7-GFP*, *pDR5::GFP*, *pDR5::GUS*, and *p35S::DII-VENUS-N7* have been described previously (Le *et al.*, 2014). Arabidopsis seeds were surface-sterilized in an aqueous solution of 30% (w/v) hydrogen peroxide and 85% (v/v) ethanol at a ratio of 1:4 (v/v). The seeds were then sown onto half-strength Murashige and Skoog (MS) medium supplemented with agar (0.8%, w/v) and sucrose (1%, w/v) and kept in the dark for 2 d at 4 °C. Seedlings were grown in a vertical orientation in growth chambers under a 16-h light / 8-h dark cycle at 22±2 °C.

### Vector construction and plant transformation

To generate the construct for complementation of *dis1-1*, a 4112-bp genomic sequence of *DIS1* was amplified by PCR with specific primers (forward primer 5′-TTGAGCTCTTTATTACC TTGAAAACAGGTCATA-3′ and reverse primer 5′-TTGGTACCC TAGAACTTAAGCTCTTGAGTGGAA-3′; *Sac* I and *Kpn* I restriction sites underlined, respectively). The PCR product was verified by DNA sequencing and cloned into the pCAMBIA1300 vector (CAMBIA). The construct was then transformed into *dis1-1* mutants. Transgenic plants were selected on half-strength MS medium containing 25 mg l^−1^ hygromycin. The T_2_ transgenic plants were used for root gravitropic experiments.

### Curvature and growth analyses

To examine root gravity responses, 4-d-old seedlings grown on vertical plates were rotated 90° and photographs were taken at selected time points after reorientation. The root growth rate was calculated using the increase in length from the start time point at reorientation. To examine hypocotyl gravity responses, 3-d-old seedlings grown vertically on half-strength MS medium in darkness were reoriented at 90° for 24h. The curvature angles and root growth were measured using ImageJ (http://rsb.info.nih.gov/ij/).

### Observation of amyloplast sedimentation

To image amyloplast movement in the central S2 columella cells (Leitz *et al.*, 2009), roots were prepared and mounted on a rotatable stage of a horizontally oriented BX51 microscope (Olympus, Japan). Differential interference images were captured at 1-s intervals for 600s after a 90° reorientation (Wang *et al.*, 2015).

### Apparent local viscosity

Using the Stokes–Einstein relation, the apparent local viscosity *η* in each subcellular region was measured using the short-time diffusion coefficient *D*_*x,y*_ = *k*_B_ *T*/2*Rη*_*x*,*y*_, where *k*_B_ is the Boltzmann constant, *T* is the temperature, and *R* is the amyloplast radius (Crocker *et al.*, 2000; Levine and Lubensky, 2000). We analyzed the Brownian motion and collective motion by measuring the short-time Brownian diffusion, the intermediate-time caged sub-diffusion, and the long-time normal diffusion of each amyloplast during sedimentation.

### Actin labeling

Imaging of actin filaments in Arabidopsis roots was performed as previously described (Le *et al.*, 2003) with slight modifications. Briefly, the roots were incubated in PME buffer (50mM PIPES, 5mM MgSO_4_, and 5mM EGTA, pH6.9) containing 300 µM m-maleimidobenzoyl-N-hydroxysuccinimide ester for 30min. The roots were then incubated in PME buffer containing 2% paraformaldehyde in PME buffer for 1h. The roots were washed three times with PME buffer and then treated with 0.1% Y-23 for 10min. Samples were further washed with PME buffer and incubated in actin-staining buffer (PME, 1% glycerol, and 0.3M mannitol and 0.1 µM Alexa Fluor 488 phalloidin) (Invitrogen, USA) at 4 ºC in the dark overnight. Images were captured using a FV1000-MPE confocal laser-scanning microscope (Olympus, Japan). To quantitatively evaluate the actin bundling in gravity-sensing cells, the skewness of the actin fluorescence intensity distribution was measured as previously described using the Skewness plug-in on ImageJ (Higaki, *et al.*, 2010).

### Latrunculin B treatment

Lat B treatment was conducted as previously described (Hou *et al.*, 2004). After seedlings grown in a vertical direction for 4 d, 200nM Lat B solution was added to the Petri dishes. After 1h treatment, the Lat B solution was removed and the Petri dishes were kept vertically for an additional 30min before the dishes were reoriented by 90°. Photographs were then taken at selected time points and the curvature of roots was measured using ImageJ.

### DII-VENUS fluorescence intensity measurement

Four-day-old DII-VENUS (a sensitive auxin input reporter) seedlings grown vertically on the surface of half-strength MS medium were transferred onto a new plate. After another 2h vertical growth, the plates were rotated 90° from the original direction, then the seedlings were mounted and the fluorescence was imaged at selected time points using a FV1000-MPE confocal laser scanning microscope (Olympus, Japan). DII-VENUS fluorescence intensity was analyzed using ImageJ as previously described (Wang *et al.*, 2015).

### FM4-64 staining and confocal microscope observation

Four-day-old seedlings were incubated in half-strength MS liquid medium containing 5 μg ml^–1^ of the membrane-selective dye FM4-64 (Invitrogen, USA) for 10min, followed by washing three times with half-strength MS liquid medium. After incubation in half-strength liquid MS medium for 20min at room temperature, the seedlings were mounted and the fluorescence was imaged using a FV1000-MPE confocal laser scanning microscope (Olympus, Japan).

### Brefeldin A (BFA) treatment

To monitor cycling of PIN proteins, 4-d-old seedlings were incubated in half-strength MS liquid medium containing 50 μM BFA for 2h, followed by 1h and 2h of washing with half-strength MS liquid medium. The seedlings were then mounted and images were captured at selected time points using a FV1000-MPE confocal laser-scanning microscope (Olympus, Japan).

## Results

### Intracellular environment of root gravity-sensing cells revealed by microrheological analysis

**Fig. 1.**
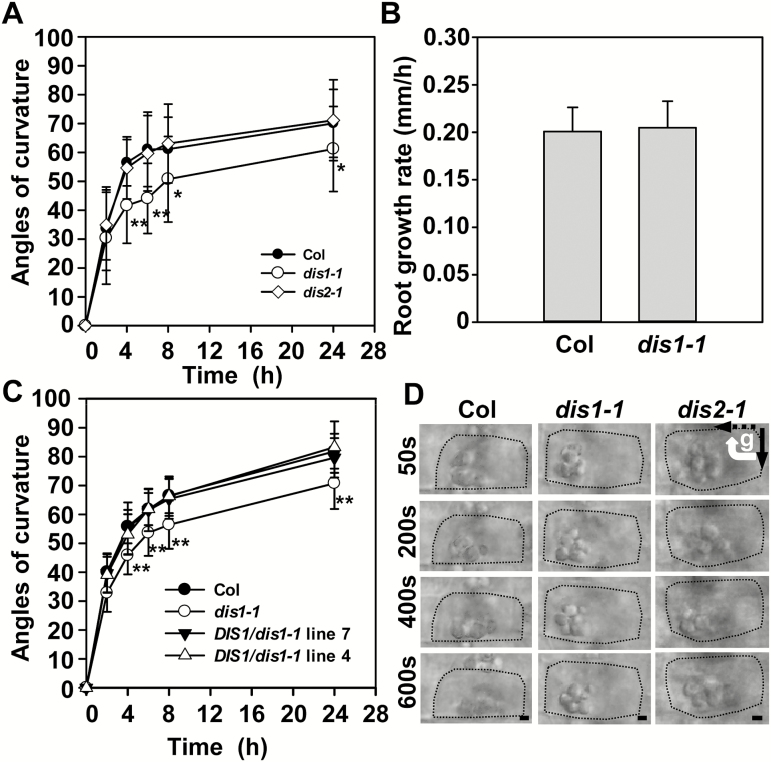
The ARP2/3 complex subunit *DIS1* mutant *dis1-1* displayed reduced root curvature and decreased amyloplast movement compared with wild-type plants. (A) Root curvature of Col, *dis1-1*, and *dis2-1* after 90° reorientation. Four-day-old seedlings were rotated by 90° to test the gravitropic response, with angles of root curvature measured at the indicated time points. Values are means ±SD (*n*=30–50 seedlings). Asterisks indicate significant differences relative to Col (Student’s *t*-test, *P<0.05, **P<0.01). (B) Root growth rates (mm h^–1^) of wild-type plants and *dis1-1* mutants. Values are means ±SD (*n*=35–45 seedlings). (C) Root curvature of Col, *dis1-1*, and two complementation lines. Values are means ±SD (*n*=20–50 seedlings). Asterisks indicate significant differences relative to Col (Student’s *t*-test, **P<0.01). (D) Time-lapse images of amyloplast sedimentation in the central columella cells of Col, *dis1-1*, and *dis2-1* after 90° reorientation. Black arrows at top-right indicate the direction of the gravity vector before (solid line) and after (dashed) reorientation. Scale bars are 2 µm.

To investigate the role of the ARP2/3 complex in root gravitropism, the *ARP3/DIS1* mutant *dis1-1* and *ARPC2A/DIS2* mutant *dis2-1* were used for root gravitropic analysis. Four-day-old seedlings grown vertically were reoriented by 90° and root curvature was measured at selected time points. As shown in Fig. 1A, the *dis1-1* mutants showed reduced root curvature compared with wild-type plants after 90° reorientation of the roots. Consistent with previous work, *dis2-1* showed similar root curvature to the wild-type plants (Reboulet *et al.*, 2010). The wild-type plants and *dis1-1* mutants showed similar root growth rates after gravity stimulation, indicating that root growth was not impaired in *dis1-1* mutants (Fig. 1B). In contrast to the different gravitropic responses found in *dis1-1* and *dis2-1* roots, these two mutants showed similar increases in hypocotyl curvature after 90° reorientation for 24h in darkness, indicating the different regulatory mechanisms of *ARP3/DIS1* and *ARPC2A/DIS2* in response to gravity stimulation between roots and shoots (Supplementary Fig. S1 at *JXB* online).

To test whether root gravity defects in *dis1-1* mutants result from *ARP3*/*DIS1* disruption, the genomic DNA sequence of *ARP3*/*DIS1* was introduced into the *dis1-1* mutants. The transgenic complementation lines can rescue *dis1-1* trichome and root gravity defects, indicating that *ARP3*/*DIS1* takes part in root gravitropism (Fig. 1C and Supplementary Fig. S2).

**Fig. 2.**
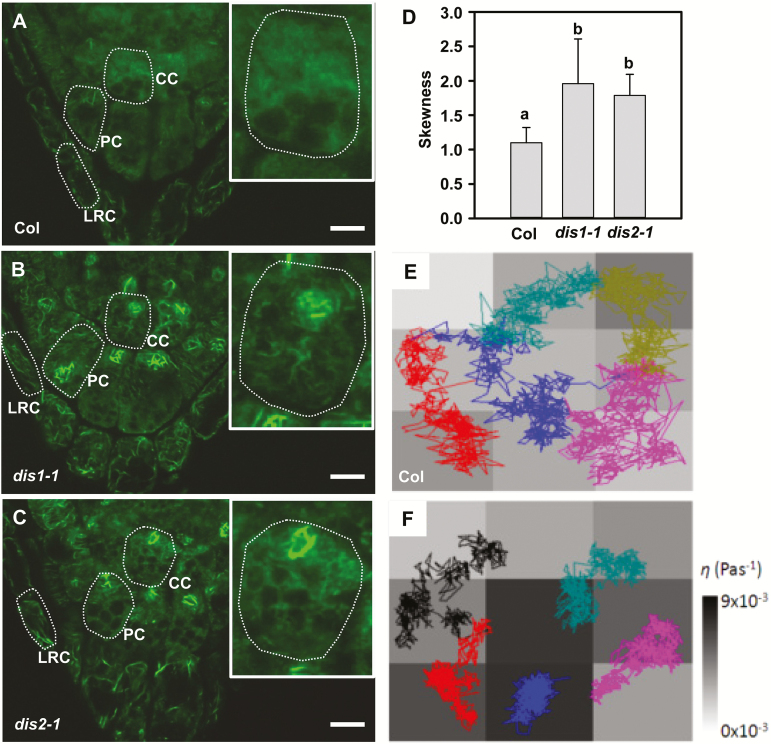
The *dis1-1* mutant showed different actin cytoskeleton organization and local apparent viscosity in the central columella cells compared with wild-type plants. (A-C) Organization of the actin cytoskeleton in the central columella cells of plants of Col (A), *dis1-1* (B), and *dis2-1* (C). Actin filaments in fixed root tips were labeled with Alexa Fluor phalloidin dyes and viewed with a confocal microscope. Inset: enlarged CC cell. The cell outline is indicated by dashed-lines. Scale bars are 10 µm. (D) Microfilament bundling (skewness) was measured in the CC cells of Col, *dis1-1*, and *dis2-1* plants. Values are means ±SD (*n*=5–9 seedlings). Different letters indicate significantly different means (one-way ANOVA test). (E, F) Movement trajectories of amyloplasts in the central columella cells after 90° reorientation and apparent local viscosity in the corresponding cells in wild-type (E) and *dis1-1* (F) plants. Polygonal lines in different shades denote different amyloplasts. (This figure is available in color at *JXB* online.)

It has been reported that mutation in *ARP3*/*DIS1* induced the formation of disorganized, thick actin bundles in developing trichome branches (Le *et al.*, 2003). Sedimentation of amyloplasts is correlated with intracellular components such as actin filaments during gravity sensing (Blancalfor, 2013). To test the influence of actin organization on amyloplast movement in CC cells of wild-type and *dis1-1* plants, the dynamic movements of amyloplasts in the CC cells were captured using time-lapse imaging after 90° reorientation. As shown in Fig. 1D, amyloplasts are initially located at the bottom of the CC cells. After reorientation for 400s, most of the amyloplasts reached the new bottom side of the CC cells. Most amyloplasts in the *dis1-1* and *dis2-1* mutants, however, stayed in the middle of the CC cells (Fig. 1D), indicating that the intracellular environment of the gravity-sensing cells in these mutants might be different from that in the wild-type plants. We then labeled actin filaments in the root cells using Alexa Fluor 488 phalloidin dyes (Le *et al.*, 2003). Differing from the formation of actin filaments/bundles in PC and LRC cells, only diffuse fluorescent signals were observed in the CC cells of wild-type plants, consistent with results reported previously (Hou *et al.*, 2004) (Fig. 2A). By contrast, the *dis1-1* and *dis2-1* mutants displayed thick actin bundles surrounding the amyloplasts in the CC cells, as well in the PC and LRC cells (Fig. 2B, C). To quantitatively evaluate the bundling of actin, the skewness of the actin fluorescence intensity distribution in CC cells was measured (Higaki, *et al.*, 2010). The significantly increased values of skewness in the *dis* mutants revealed that the formation of actin bundles may alter the amyloplast kinetics in the CC cells (Fig. 2D).

Microrheology has been rapidly developed as a powerful method to explore the relationships between local mechanical responses and local structures in inhomogeneous fluids (Wirtz, 2009). The amyloplasts in the columella cells can be used as native microprobes to analyze the inhomogenous intracellular environment. Recently, we implemented a novel method for measurement of diffusive dynamics and *in planta* microrheological analysis of amyloplasts by multi-particle tracking in the CC cells of Arabidopsis root caps (Zheng *et al.*, 2015). We found that actin organization dominated the intracellular environment of CC cells and highlighted the spatial heterogeneity and the cage-confinement of amyloplasts characterized by the local apparent viscosity, *η* (Zheng *et al.*, 2015). Here, we plotted the spatial coupling of the movement trajectories of each amyloplast and the local viscosity in corresponding sub-regions of columella cells (Fig. 2E, F). In the wild-type plants, amyloplast trajectories showed a frequent small-step rattling motion with an occasional large-step chain-like motion. The confined Brownian motion within cages coincides with high local viscosity (dark gray) regions, while the co-operative out-of-cage motion of several amyloplasts emerges in low local viscosity (light gray) regions (Fig. 2E). By contrast, in the *dis1-1* mutant, the amyloplasts rattled randomly within separate cages but did not undergo a co-operative cage escape (Fig. 2F). This indicates a stronger cage confinement that can be characterized by the greatly increased local viscosity and its spatial fluctuation. In each type of CC cells, the compact and loose trajectories of amyloplasts respectively correspond to the higher and lower local viscosity in that sub-region (Fig. 2E, F). Taken together, this microrheological analysis indicates that the actin cytoskeleton functions in affecting amyloplast movements through regulating local viscosity in the CC cells.

### ARP3/DIS1 is also required for gravity signal transduction

**Fig. 3.**
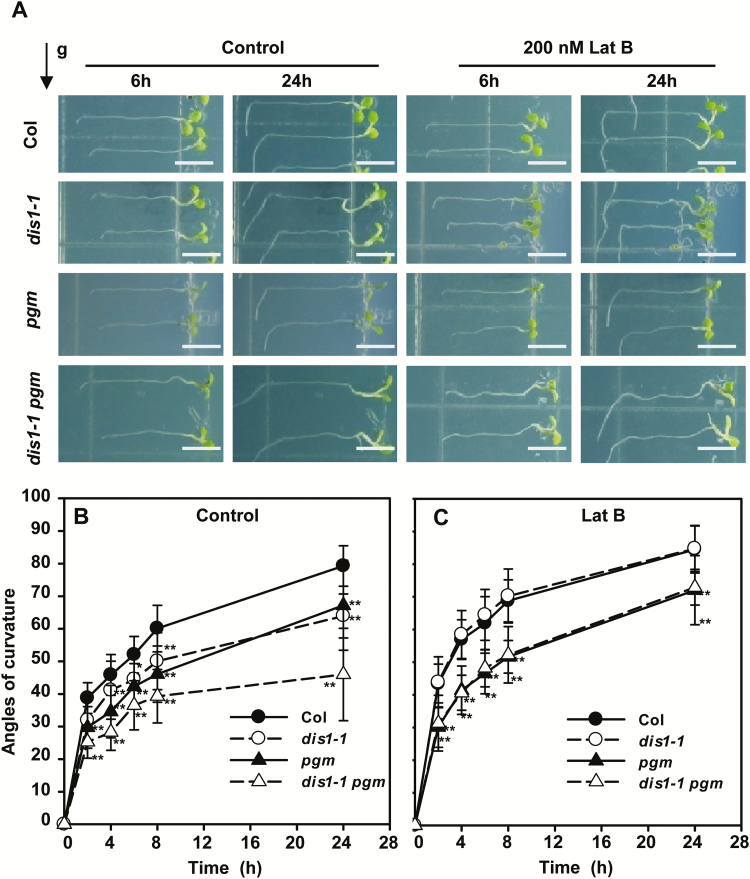
Root curvature of Col, *dis1-1*, *pgm*, and *dis1-1 pgm* seedlings. (A) Images of root curvature of untreated and Lat B-treated Col, *dis1-1*, *pgm*, and *dis1-1 pgm* seedlings after gravity stimulation. Four-day-old seedlings were rotated by 90° to test their gravitropic response. Images were taken 6h and 24h after reorientation. The black arrow at the top-left indicates the direction of the gravity vector after reorientation. Scale bars are 0.5cm. (B, C) Quantification of root gravitropic response in Col, *dis1-1*, *pgm*, and *dis1-1 pgm* seedlings before and after treatment with 200nM Lat B for 1h. Four-day-old seedlings were rotated by 90° to test their gravitropic response. Values are means ±SD (*n*=20–40 seedlings). Asterisks indicate significant differences relative to Col (Student’s *t*-test, **P<0.01). (This figure is available in color at *JXB* online.)

In addition to its role in gravity sensing, we then questioned whether ARP3/DIS1 functions in gravity signal transduction. The formation of starch-filled amyloplasts, the statoliths, is very important for gravity sensing (Sack, 1997). PHOSPHOGLYCERATE/BISPHOSPHOGLYCERATE MUTASE (PGM) is involved in starch biosynthesis. The starchless *pgm* mutant exhibits a delayed gravitropic response in roots (Caspar and Pickard, 1989; Kiss *et al.*, 1989). To investigate whether *dis1-1* takes part in both gravity sensing and gravity signal transduction, *dis1-1 pgm* double-mutants were generated to examine their root gravitropic responses. As shown in Fig. 3A and B, *dis1-1* and *pgm* single-mutant roots showed similar root gravitropic defects, whereas *dis1-1 pgm* double-mutants displayed much stronger root gravitropic defects than the single-mutants. When treated with Lat B, the Col and *dis1-1* seedlings showed a similar enhanced bending response, indicating that breaking down of the actin network in the root caps can rescue root gravitropic defects in the *dis1-1* mutant (Fig. 3C). This enhanced gravitropic response in Lat B-treated roots was reduced in both *pgm* and *dis1-1 pgm* mutants (Fig. 3C). Together with amyloplast movement, this indicates that ARP3/DIS1 takes part in both gravity sensing and gravity signal transduction phases.

### Asymmetric auxin redistribution in dis1-1 root tips is delayed during the gravitropic response

**Fig. 4.**
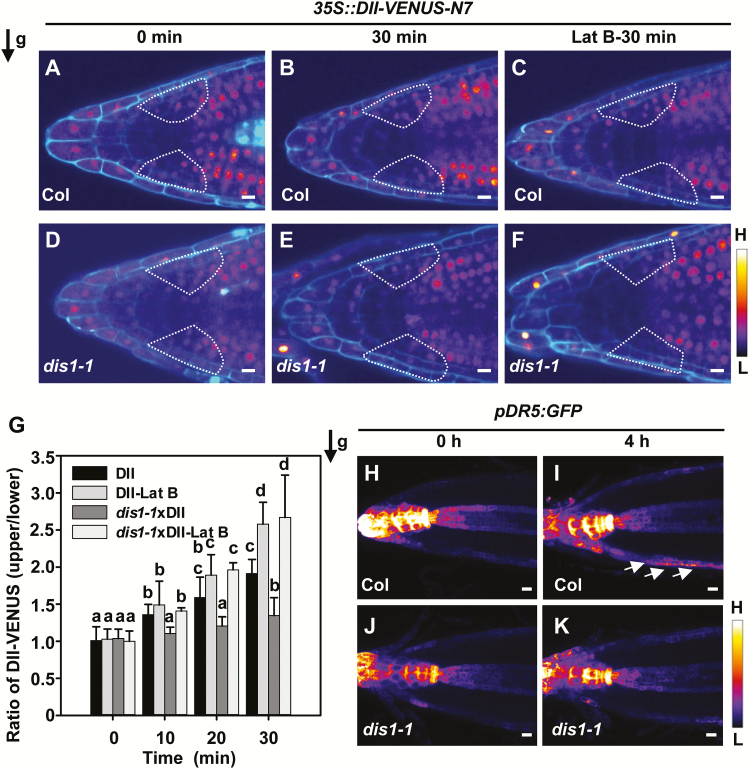
The *dis1-1* mutant showed decelerated asymmetric auxin distribution between the upper and lower side of the root tip following a 90° gravity stimulation. (A–F) Representative heat map images of DII-VENUS fluorescence show the asymmetric distribution of DII-VENUS fluorescence in the root tips of wild-type and *dis1-1* plants after 90° reorientation at the indicated time points. Cell outlines were visualized after staining with propidium iodide. The black arrow at the top-left indicates direction of the gravity vector after reorientation. The bar on the lower-right indicates the signal intensity range from high (H) to low (L). Scale bars are 10 µm. (G) The fold-changes in DII-VENUS ratios between wild-type and *dis1-1* following a 90° gravity stimulation at the indicated time points. Values are means ±SD (*n*=3–9 seedlings). Different letters indicate significantly different means (one-way ANOVA test). (H–K) Heat map images of *pDR5::GFP* show the asymmetric distribution of *pDR5::GFP* in root tips of wild-type and *dis1-1* plants after 90° reorientation at the indicated time points. The arrows indicate the distribution of *pDR5::GFP* expression on the lower side of the root caps. The direction of the gravity vector after reorientation is indicated at the top-left. The bar on the lower-right indicates the signal intensity range from high (H) to low (L). Scale bars are 10 µm. (This figure is available in color at *JXB* online.)

During gravity signal transduction, asymmetric auxin redistribution between the upper and lower side of root tips can cause differential root growth and lead to root curvature (Band *et al.*, 2012). To test whether *DIS1* regulates asymmetric redistribution of auxin after the gravitropic response, a sensitive auxin input reporter, DII-VENUS, was introduced to monitor the speed and magnitude of changes in auxin distribution during the root gravitropic response (Brunoud *et al.*, 2012). Gravity-induced rapid auxin redistribution to the lower side of the root tips occurred within minutes of a 90° gravity stimulation. In the root tips, cells within the lateral root cap mediate the creation of shootward auxin fluxes (Ottenschläger *et al.*, 2003; Swarup *et al.*, 2005). As shown in Fig. 4A and B, the DII-VENUS signal was reduced in LRC cells on the lower side of root tip after a 90° gravity stimulation for 30min. This asymmetric auxin redistribution between the upper and lower sides of the root tip was reduced in the *dis1-1* mutant (Fig. 4D, E). When treated with Lat B, both wild-type and *dis1-1* plants showed increased asymmetric auxin redistribution in the root tips (Fig. 4C, F). Auxin asymmetry was quantitatively analyzed by measuring the DII-VENUS signal ratios between the upper and the lower sides of LRC cells adjacent to the columella cells. In wild-type roots, the DII-VENUS ratios continued to increase after 90° gravity stimulation and were approximately two-fold higher after 30min. However, increases in the DII-VENUS ratios were significantly smaller in *dis1-1* plants. Disruption of the actin cytoskeleton with Lat B significantly increased DII-VENUS ratios in wild-type plants and *dis1-1* mutants relative to untreated plants (Fig. 4G). We also monitored the expression patterns of an auxin activity reporter, *pDR5::GFP*, in wild-type and *dis1-1* root tips after gravity stimulation. Analysis of the *pDR5::GFP* expression pattern showed that stronger GFP signals were found on the lower side of root tips in the wild-type compared with the *dis1-1* mutant after 4h of gravity stimulation (Fig. 4H–K). Analysis of *pDR5::GUS* expression patterns during gravity stimulation also showed similar results to *pDR5::GFP* (Supplementary Fig. S3).

In addition, we tested whether exogenous auxin (IAA or NAA) can rescue *dis1-1* root gravitropic defects. Three-day-old seedlings were transferred to half-strength MS medium containing IAA (1 or 10nM) or NAA (1 or 10nM) for 18h. The root curvature of wild-type and *dis1-1* plants was then measured at 4h and 24h after 90° reorientation. As shown in Supplementary Fig. S4, neither IAA nor NAA could rescue the reduced root curvature in the *dis1-1* mutants. These findings showed that the delayed root gravitropic response in *dis1-1* is not caused by decreased overall auxin accumulation, and that ARP3/DIS1 may regulate the root gravitropic response by affecting polar auxin transport.

### Vesicle trafficking is defective in dis1-1 mutants

**Fig. 5.**
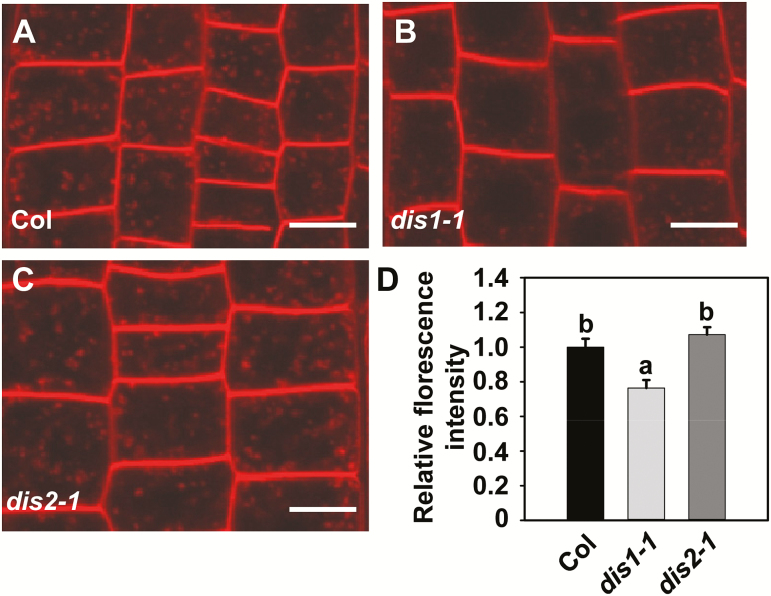
*DIS1* deficiency caused decelerated vesicle trafficking. (A–C) Vesicle trafficking was suppressed in *dis1-1* compared with Col and *dis2-1* plants. Roots of 4-d-old seedlings stained with FM4-64 (5 µg ml^–1^, 30min) were observed under a confocal microscope. Scale bars are 10 µm. (D) Relative FM4-64 internalization fluorescence intensity in Col, *dis1-1*, and *dis2-1* cells. Values are means ±SD (*n*=20–22 cells). Different letters indicate significantly different means (one-way ANOVA test). (This figure is available in color at *JXB* online.)

The ARP2/3 complex has previously been shown to be important for actin filament assembly and is needed for cell motility, vesicle trafficking, and endocytosis (Rotty *et al.*, 2013). It has been reported that in Arabidopsis PIN proteins undergo constitutive endocytic recycling between the plasma membrane and the endosomal compartments (Kleine-Vehn *et al.*, 2010). It was hypothesized that ARP3/DIS1 might regulate auxin transport by affecting vesicle trafficking (Reboulet *et al.*, 2010). FM4-64 is a water-soluble marker that is widely used to study endocytosis, vesicle trafficking, and organelle organization in living eukaryotic cells (Bolte *et al.*, 2004). We therefore used FM4-64 to monitor endocytosis in root epidermal cells of wild-type, *dis1-1*, and *dis2-1* plants. As shown in Fig. 5A, after 30min of staining, the FM4-64 dye was internalized and substantial numbers of punctuated fluorescent vesicles were detected in the cytosol of wild-type root epidermal cells. Conversely, only a few fluorescent vesicles were observed in the root epidermal cells of *dis1-1* mutants, indicating that endocytosis is defective in the *dis1-1* mutant (Fig. 5B). The *dis2-1* mutants showed a similar result to the wild-type plants after FM4-64 staining (Fig. 5C). Quantification of FM4-64 uptake showed a significantly decreased uptake of FM4-64 in *dis1-1* mutants compared with wild-type plants and *dis2-1* mutants (Fig. 5D). These results suggest that ARP3/DIS1 positively regulates endocytosis.

### ARP3/DIS1 mutation causes decelerated PIN cycling

**Fig. 6.**
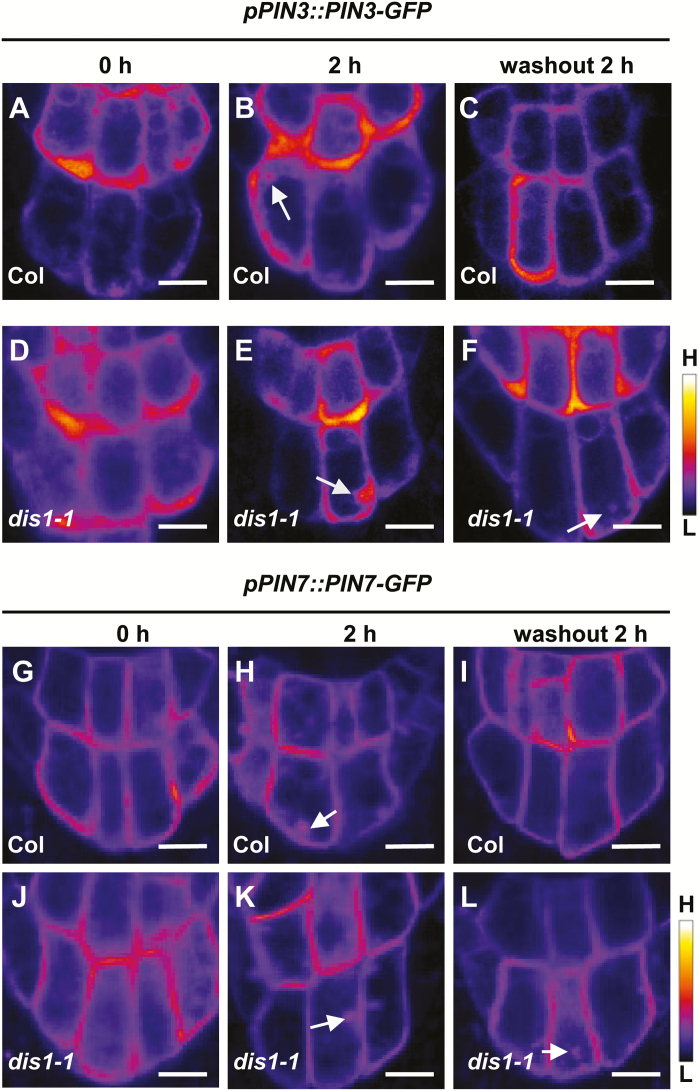
Intracellular cycling of PIN3 and PIN7 is decelerated in the *dis1-1* mutants. (A–F) Heat map images of *pPIN3::PIN3-GFP* fluorescence in 4-d-old root cells of wild-type (A–C) and *dis1-1* (D–F) seedlings after treatment with 50 µM BFA and subsequent wash-out. The arrows indicate BFA bodies. The bar on the lower-right indicates the signal intensity range from high (H) to low (L). Scale bars are 10 µm. (G–L) Heat map images of *pPIN7::PIN7-GFP* fluorescence in 4-d-old root cells of wild-type (G–I) and *dis1-1* (J–L) seedlings after treatment with 50 µM BFA and subsequent wash-out. The arrows indicate BFA bodies. The bar on the lower-right indicates the signal intensity range from high (H) to low (L). Scale bars are 10 µm. (This figure is available in color at *JXB* online.)

During gravity signal transduction, polar auxin transport is affected by PIN proteins (Křeček *et al.*, 2009). The actin cytoskeleton takes part in auxin transporter endocytosis and cycling by regulating vesicular trafficking (Zhu and Geisler, 2015). In Arabidopsis, PIN3 and PIN7 have been shown to localize to the columella cells and to exhibit constitutive intracellular cycling between the plasma membrane and endosomal compartments (Kleine-Vehn *et al.*, 2010). The fungal toxin BFA, a vesicle transport inhibitor that can inhibit protein trafficking in the endomembrane system to form BFA compartments, has been used to investigate PIN trafficking (Nebenführ *et al.*, 2002; Chen *et al.*, 2012; Lin *et al.*, 2012). To determine whether DIS1 regulates auxin transport by affecting PIN3 and PIN7 cycling in the columella cells, *pPIN3::PIN3-GFP* and *pPIN7::PIN7-GFP* lines were introduced into the *dis1-1* mutant background. After 2h of BFA treatment, PIN3-GFP and PIN7-GFP aggregated into BFA bodies in the columella cells of both wild-type plants and the *dis1-1* mutants (Fig. 6B, E, H, K). For PIN3, as BFA has a stronger effect on the intracellular PIN3 trafficking in gravity-stimulated roots than on non-stimulated roots (Kleine-Vehn *et al.*, 2010), there was no obvious difference in accumulated PIN3-GFP in BFA bodies between the wild-type and the *dis1-1* mutant. After 2h of BFA wash-out, normal plasma membrane localization of PIN3-GFP or PIN7-GFP was recovered in the wild-type plants (Fig. 6C, I). However, aggregated GFP fluorescence remained in the *dis1-1* mutants, indicating that PIN3 and PIN7 recycling to the plasma membrane in the columella cells is regulated by ARP3/DIS1 (Fig. 6F, L).

**Fig. 7.**
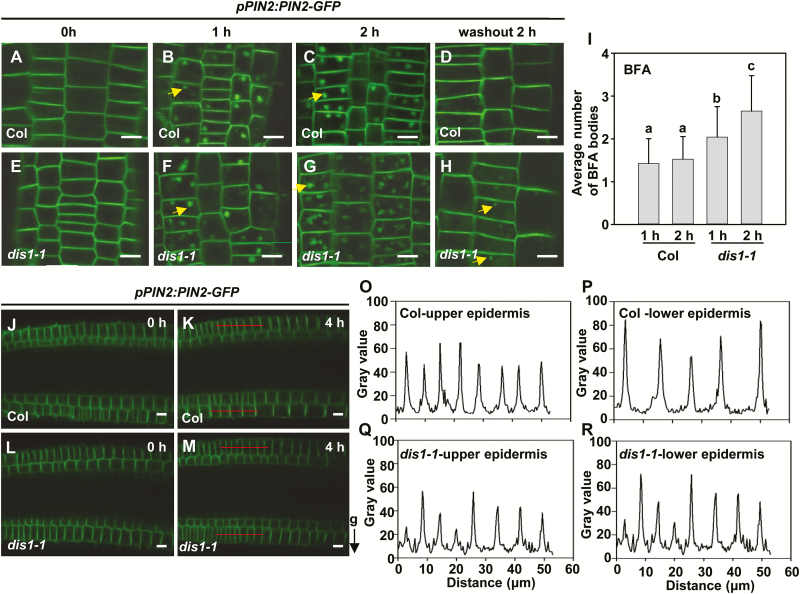
PIN2 cycling is decelerated in *dis1-1* mutants compared with wild-type plants. (A–H) Internalization of PIN2 between Col and *dis1-1* plants after treatment with 50 µM BFA and subsequent wash-out. The arrows indicate BFA bodies. Scale bars are 10 µm. (I) Quantification of BFA bodies in Col and *dis1-1* plants. Values are means ±SD (*n*=40*–*90 cells). Different letters indicate significantly different means (one-way ANOVA test). (J–M) Observation of fluorescence of *pPIN2::PIN2-GFP* in 4-d-old root cells of wild-type (J, K) and *dis1-1* (L, M) seedlings following the gravity stimulation. The arrow on the lower-right indicates direction of the gravity vector after reorientation. Scale bars are 10 µm. (O–R) Quantification of *pPIN2::PIN2-GFP* fluorescence intensity at the plasma membrane in the upper and lower epidermis of roots of wild-type plants (O, P) and *dis1-1* mutants (Q, R). The *pPIN2::PIN2-GFP* fluorescence intensity is calculated along the lines shown in (K) and (M). (This figure is available in color at *JXB* online.)

PIN2 is localized to the apical end of epidermal cells and the basal end of cortical cells in the root tips and is critical for the root gravitropic response by regulating auxin redistribution. Dynamic changes in PIN2 proteins are also important for auxin flows during the root gravitropic response (Chen *et al.*, 1998; Müller *et al.*, 1998; Rahman *et al.*, 2010; Lin *et al.*, 2012; Sassi *et al.*, 2012; Rigó *et al.*, 2013). Polar localization and expression of PIN2 were not affected in the *dis1-1* mutants compared with wild-type plants under normal growth conditions (Fig. 7A, E). After 1h of BFA treatment, the accumulation of PIN2-containing BFA bodies increased in the *dis1-1* mutants compared with wild-type plants (Fig. 7B, F, I). This effect was amplified after 2h of BFA treatment (Fig. 7C, G, I). After 2h of BFA wash-out, almost all the BFA bodies disappeared and normal polar localization of PIN2-GFP was detected in the epidermal cells of wild-type plants (Fig. 7D). Conversely, small numbers of BFA bodies still accumulated in the epidermal cells of *dis1-1* plants, indicating that accumulated PIN2-GFP bodies were not recovered effectively (Fig. 7H). As for the *dis2-1* mutant, there was no difference in the accumulation of PIN2-containing BFA bodies between wild-type plants and the *dis2-1* mutants (Supplementary Fig. S5), indicating that ARP3/DIS1 and ARPC2/DIS2 may play different roles in regulating PIN protein cycling. When the actin cytoskeleton organization was analyzed in the epidermal cells of the root transition zone where PIN2 is localized, no obvious differences were found among Col, *dis1-1*, and *dis2-1* (Supplementary Fig. S6).

Next, we examined the redistribution of PIN2-GFP in wild-type and *dis1-1* plants after gravity stimulation. After 90° reorientation of root tips for 4h, PIN2-GFP signals in wild-type plants were higher on the lower side of the root tip than on the upper side, with the difference being pronounced in the epidermal cells (Fig. 7J, K, O, P). By contrast, PIN2-GFP signals in *dis1-1* roots were not significantly different between the upper and lower sides of the epidermal cells (Fig. 7L, M, Q, R). These results indicate that ARP3/DIS1 participates in PIN2 internalization and recycling between the plasma membrane and endosomal compartments in the root tips.

## Discussion

The actin cytoskeleton has been proposed to be an important component of gravity sensing and signal transduction, with pharmacological and genetic evidence beginning to reveal the roles of the actin cytoskeleton in root gravitropism (Blancaflor, 2013). In this study, we provide evidence that the ARP2/3 complex subunit ARP3/DIS1 is involved in the root gravity response by affecting both amyloplast sedimentation and PIN-mediated polar auxin transport.

### Microrheological analysis provides new insights by revealing the role of the actin cytoskeleton in gravity sensing

Although the behavior of amyloplast sedimentation in gravity-sensing cells has been investigated intensively (Sack *et al.*, 1986; Yoder *et al.*, 2001; Saito *et al.*, 2005; Leitz *et al.*, 2009; Nakamura *et al.*, 2011; Toyota *et al.*, 2013), the effects of intracellular components, including vacuoles and the actin cytoskeleton, on amyloplast movement have not been characterized in detail (Saito *et al.*, 2005; Nakamura *et al.*, 2011). In this study, pharmacological treatments that disrupt the actin filaments induced a rapid and free diffusive sedimentation of amyloplasts, while the actin bundles that formed in actin mutants caused restrained sedimentation of amyloplasts (Fig. 1D and Fig. 2). Consistent with these results, pharmacological treatments that disrupt the actin filaments have previously been shown to induce an enhanced gravity sensitivity in roots, hypocotyls, and inflorescence stems (Yamamoto and Kiss, 2002; Hou *et al.*, 2003, 2004; Nakamura *et al.*, 2011). Thus, in contrast to the formation of actin bundles in other cells, the fine actin filament network in the root CC cells may provide a suitable intracellular environment for the unimpeded sedimentation of amyloplasts (Blancaflor, 2013).

Furthermore, our microrheological analysis revealed the compact and loose trajectories of amyloplasts corresponded to higher and lower local apparent viscosity in different subregions of the central columella cells, respectively (Fig. 1D and Fig. 2)

### ARP3/DIS1 plays an important role during root gravity sensing and signal transduction

Sedimentation of amyloplasts in the columella cells provides the means for converting the gravitational potential energy into a biochemical signal (Leitz *et al.*, 2009). Sedimentation of amyloplasts onto the lower side of the columella cells can trigger the formation of the lateral auxin gradient. In our study, the *dis1-1 pgm* double-mutants showed strong gravitropic defects in roots compared with single-mutants, supporting the proposed role for ARP3/DIS1 in gravity signal transduction in addition to gravity perception (Fig. 3). Asymmetric auxin redistribution between the upper and lower side of the root tips was reduced in *dis1-1* mutants relative to that in wild-type plants after gravity stimulation (Fig. 4), and analysis showed that ARP3/DIS1 mediates vesicle trafficking during gravity signal transduction (Fig. 5). These results further confirm the role of APR3/DIS1 in root signal transduction through regulating auxin polar transport.

### ARP3/DIS1 is involved in PIN-mediated auxin transport

Cross-talk between polar auxin transport and the actin cytoskeleton in plant development has been the subject of discussion (Zhu and Geisler, 2015). Polar auxin transport is mediated by specific auxin influx and efflux carriers, with the auxin efflux carrier PIN family reported to be important for polar auxin transport during gravitropism (Swarup *et al.*, 2005; Křeček *et al.*, 2009).

PIN proteins redirect the auxin flows, representing an important response mechanism to gravity stimulation (Kleine-Vehn *et al.*, 2010; Baster *et al.*, 2013). The abundance and localization of PIN proteins at the plasma membrane are finely regulated and controlled by transcriptional regulation (Blilou *et al.*, 2005; Cui *et al.*, 2013; Garay-Arroyo *et al.*, 2013; Wang *et al.*, 2015), phosphorylation regulation (Sukumar *et al.*, 2009; Huang *et al.*, 2010; Ganguly *et al.*, 2012), or degradation (Kleine-Vehn *et al.*, 2008; Sassi *et al.*, 2012). PIN protein trafficking between the plasma membrane and intracellular compartments is also crucial for root gravitropism (Lin *et al.*, 2012; Mei *et al.*, 2012). It has been reported that actin filaments regulate vesicle trafficking and cycling of PINs, such as PIN1 (Geldner *et al.*, 2001), PIN2 (Chen *et al.*, 2012; Lin *et al.*, 2012), and PIN3 (Friml *et al.*, 2002; Harrison and Masson, 2008). Our results provide evidence that ARP3/DIS1 is also important for PIN trafficking and cycling during root gravitropism. *dis1-1* mutants showed reduced FM4-64 dye uptake, indicating that *ARP3/DIS1* may function in PIN internalization (Fig. 5). BFA treatment and wash-out experiments indicated that the cycling of PIN2, PIN3, and PIN7 is dependent on the function of ARP3/DIS1 (Figs 6 and 7). These results indicated that ARP3/DIS1-mediated actin organization also participates in asymmetric auxin redistribution by regulating PIN internalization and recycling.

### The roles of ARP3/DIS1 and ARPC2A/DIS2 in root gravitropism

Previous reports have indicated that the *dis1-1* and *dis2-1* mutants show similar defects in trichomes and hypocotyls (Le *et al.*, 2003; El-Assal *et al.*, 2004; Basu *et al.*, 2005); however, they display different responses to gravitropism and phototropism (Reboulet *et al.*, 2010). In the CC cells of root tips, *dis1-1* and *dis2-1* mutants show similar actin bundling and amyloplasts sedimentation, indicating that both ARP3/DIS1 and APRC2A/DIS2 may contribute to the function of ARP2/3 during the gravity sensing phase.

In *Saccharomyces cerevisiae*, analyses of actin nucleation activity, cell growth, and endocytosis of different p35/ARPC2 mutant alleles showed that the measured loss of the actin nucleation activity does not perfectly match the severity of cell growth and endocytosis defects. For example, the Surface III of ARPC2 is essential for endocytosis but not actin nucleation (Daugherty and Goode, 2008). It was reported that the *dis2-1* mutation caused the accumulation of two mis-spliced transcripts that encode two proteins, dis2-1U and dis2-1S. The dis2-1S protein can interact weakly with ARPC4, indicating that the remaining dis2-1S protein in *dis2-1* may still function in some aspects of cellular function (El-Assal *et al.*, 2004). In this study, vesicle trafficking and BFA treatments showed that vesicle trafficking and PIN2 cycling were not affected in the *dis2-1* mutant, indicating that mutation in *dis2-1* may have effects on actin cytoskeleton organization but not on endocytosis and vesicle trafficking (Fig. 5 and Supplementary Fig. S5).

Previous studies have reported that *PIN3* and *PIN7* have partially overlapping expression patterns in the columella cells and function redundantly in the gravitropic response. Roots of the *pin3* mutant only show marginal defects in response to gravity stimulation. The gravitropic response defects of *pin3 pin7* double-mutant seedlings were stronger than those of either single-mutant (Kleine-Vehn *et al.*, 2010). *PIN2* single-mutants, however, display strong root gravitropic response defects (Rahman *et al.*, 2010). These two PINs, therefore, contribute differently to the gravitropic response. Moreover, it has been reported that light plays an essential role in PIN2 intracellular trafficking, probably by modulating the activity of the actin cytoskeleton (Laxmi *et al.*, 2008; Sassi *et al.*, 2012; Wan *et al.*, 2012). It was previously observed that *dis1-1* showed more severe gravitropic defects in the dark than *dis2-1* mutants (Reboulet *et al.*, 2010), suggesting the involvement of APR3/DIS1 in PIN cycling. However, it will be worth checking whether ARPC2A/DIS2 has impacts on PIN2 localization in the dark. As PIN2 cycling is not dependent on ARPC2A/DIS2, the defects in root gravitropic response of *dis2-1* may be weak and, therefore, difficult to observe, in spite of the amyloplast sedimentation defects found in the *dis2-1* mutants (Fig. 1D). Unlike its behavior in roots, PIN3 plays a major role in hypocotyl gravitropism. Gravity induces the translocation of PIN3 in endodermal cells and results in an on-site auxin asymmetry across hypocotyls. In contrast to the weak gravitropic phenotype in roots, *pin3* hypocotyls display a pronounced defective gravitropic bending (Rakusová *et al.*, 2011). Interestingly, both the hypocotyls of *dis1-1* and *dis2-1* mutants displayed a similar gravitropic bending (Supplementary Fig. S1). Thus we propose that both ARP3/DIS1 and ARPC2A/DIS2 may contribute to the PIN3 translocation in shoot endodermal cells as well.

In mammals, the ARPC1 and ARPC5 subunits are each encoded by two genes. ARPC1B and ARPC5L are significantly better at promoting actin assembly than subunits with ARPC1A and ARPC5, revealing that distinctive ARP2/3 complexes (consisting of different subunit isoforms) may exert fundamentally divergent activities in higher eukaryotes (Abella *et al.*, 2016). In Arabidopsis, as well in rice, there are only one *ARPC1* and one *ARPC5*, but ARPC2 is encoded by two genes, *ARPC2A*/*DIS2* and *ARPC2B* (Le *et al.*, 2003; El-Assal *et al.*, 2004). Differing from ARPC2A/DIS2, ARPC2B has a candidate calmodulin-binding domain within its C-terminal extension (El-Assal *et al.*, 2004). Since gravistimulation can induce a transient change of cytoplasmic free calcium ion concentration, it is possible that ARPC2B is involved in root gravitropism through a calcium/calmodulin signaling pathway. One possibility is that ARPC2A/DIS2 and ARPC2B paralogs may have evolved distinct functions in regulating actin organization and endocytosis/vesicle trafficking. We then speculate that ARPC2A/DIS2 and ARPC2B may play complementary roles during gravity perception (amyloplast movement) and signal transduction (PIN protein recycling and calcium signaling), respectively.

In summary, our data indicate that the actin-related protein ARP3/DIS1 functions in root gravitropism, affecting amyloplast sedimentation in gravity perception, and mediating PIN protein cycling mainly through vesicle trafficking, thereby determining polar auxin transport in root gravity signal transduction. However, it remains to be determined if other components of the ARP2/3 complex as well the upstream WAVE/SCAR complex are involved in gravitropism and how PIN recycling is affected.

## Supplementary data

Supplementary data are available at *JXB* online.

Figure S1. Hypocotyl gravitropic responses of Col, *dis1-1*, and *dis2-1* plants.

Figure S2. Phenotype complementation of the *dis1-1* mutant.

Figure S3. Asymmetric distribution of *pDR5::GUS* between the upper and lower sides of root tips after 90° reorientation.

Figure S4. Exogenous IAA or NAA did not recover defects of root gravitropic response in *dis1-1* mutants.

Figure S5. PIN2 cycling in *dis2-1* and wild-type plants.

Figure S6. Actin cytoskeleton organization in the root transition zone of wild-type, *dis1-1*, and *dis2-1* plants.

Supplementary Data

## References

[CIT0001] Abella JV Galloni C Pernier J Barry DJ Kjaer S Carlier MF Way M 2016 Isoform diversity in the Arp2/3 complex determines actin filament dynamics. Nature Cell Biology 18, 76–86. 26655834 10.1038/ncb3286

[CIT0002] Band LR Wells DM Larrieu A 2012 Root gravitropism is regulated by a transient lateral auxin gradient controlled by a tipping-point mechanism. Proceedings of the National Academy of Sciences, USA 109, 4668–4673. 10.1073/pnas.1201498109 PMC3311388 22393022

[CIT0003] Baster P Robert S Kleine-Vehn J Vanneste S Kania U Grunewald W De Rybel B Beeckman T Friml J 2013 SCF^TIR1/AFB^-auxin signalling regulates PIN vacuolar trafficking and auxin fluxes during root gravitropism. The EMBO Journal 32, 260–274. 23211744 10.1038/emboj.2012.310 PMC3553380

[CIT0004] Basu D Le J El-Essal Sel D Huang S Zhang C Mallery EL Koliantz G Staiger CJ Szymanski DB 2005 DISTORTED3/SCAR2 is a putative Arabidopsis WAVE complex subunit that activates the Arp2/3 complex and is required for epidermal morphogenesis. The Plant Cell 17, 502–524. 15659634 10.1105/tpc.104.027987 PMC548822

[CIT0005] Basu D Le J Zakharova T Mallery EL Szymanski DB 2008 A SPIKE1 signaling complex controls actin-dependent cell morphogenesis through the heteromeric WAVE and ARP2/3 complexes. Proceedings of the National Academy of Sciences, USA 105, 4044–4049. 10.1073/pnas.0710294105 PMC2268813 18308939

[CIT0006] Blancaflor EB 2002 The cytoskeleton and gravitropism in higher plants. Journal of Plant Growth Regulation 21, 120–136. 12024227 10.1007/s003440010041

[CIT0007] Blancaflor EB 2013 Regulation of plant gravity sensing and signaling by the actin cytoskeleton. American Journal of Botany 100, 143–152. 23002165 10.3732/ajb.1200283

[CIT0008] Blancaflor EB Masson PH 2003 Plant gravitropism. Unraveling the ups and downs of a complex process. Plant Physiology 133, 1677–1690. 14681531 10.1104/pp.103.032169 PMC1540347

[CIT0009] Blilou I Xu J Wildwater M Willemsen V Paponov I Friml J Heidstra R Aida M Palme K Scheres B 2005 The PIN auxin efflux facilitator network controls growth and patterning in *Arabidopsis* roots. Nature 433, 39–44. 15635403 10.1038/nature03184

[CIT0010] Bolte S Talbot C Boutte Y Catrice O Read ND Satiat-Jeunemaitre B 2004 FM-dyes as experimental probes for dissecting vesicle trafficking in living plant cells. Journal of Microscopy 214, 159–173. 15102063 10.1111/j.0022-2720.2004.01348.x

[CIT0011] Brunoud G Wells DM Oliva M 2012 A novel sensor to map auxin response and distribution at high spatio-temporal resolution. Nature 482, 103–106. 22246322 10.1038/nature10791

[CIT0012] Caspar T Pickard BG 1989 Gravitropism in a starchless mutant of *Arabidopsis*: implications for the starch-statolith theory of gravity sensing. Planta 177, 185–197. 11539758

[CIT0013] Chen R Hilson P Sedbrook J Rosen E Caspar T Masson PH 1998 The *Arabidopsis thaliana AGRAVITROPIC 1* gene encodes a component of the polar-auxin-transport efflux carrier. Proceedings of the National Academy of Sciences, USA 95, 15112–15117. 10.1073/pnas.95.25.15112 PMC24584 9844024

[CIT0014] Chen X Naramoto S Robert S Tejos R Löfke C Lin D Yang Z Friml J 2012 ABP1 and ROP6 GTPase signaling regulate clathrin-mediated endocytosis in *Arabidopsis* roots. Current Biology 22, 1326–1332. 22683261 10.1016/j.cub.2012.05.020

[CIT0015] Crocker JC Valentine MT Weeks ER Gisler T Kaplan PD Yodh AG Weitz DA 2000 Two-point microrheology of inhomogeneous soft materials. Physical Review Letters 85, 888–891. 10991424 10.1103/PhysRevLett.85.888

[CIT0016] Cui D Zhao J Jing Y Fan M Liu J Wang Z Xin W Hu Y 2013 The *Arabidopsis* IDD14, IDD15, and IDD16 cooperatively regulate lateral organ morphogenesis and gravitropism by promoting auxin biosynthesis and transport. PLoS Genetics 9, e1003759. 24039602 10.1371/journal.pgen.1003759 PMC3764202

[CIT0017] Daugherty KM Goode BL 2008 Functional surfaces on the p35/ARPC2 subunit of Arp2/3 complex required for cell growth, actin nucleation, and endocytosis. Journal of Biological Chemistry 283, 16950–16959. 18381280 10.1074/jbc.M800783200 PMC2423265

[CIT0018] El-Assal SE-D Le J Basu D Mallery EL Szymanski DB 2004 *DISTORTED2* encodes an ARPC2 subunit of the putative *Arabidopsis* ARP2/3 complex. The Plant Journal 38, 526–538. 15086808 10.1111/j.1365-313X.2004.02065.x

[CIT0019] Friml J Wisniewska J Benkova E Mendgen K Palme K 2002 Lateral relocation of auxin efflux regulator PIN3 mediates tropism in *Arabidopsis* . Nature 415, 806–809. 11845211 10.1038/415806a

[CIT0020] Ganguly A Lee S-H Cho H-T 2012 Functional identification of the phosphorylation sites of Arabidopsis PIN-FORMED3 for its subcellular localization and biological role. The Plant Journal 71, 810–823. 22519832 10.1111/j.1365-313X.2012.05030.x

[CIT0021] Garay-Arroyo A Ortiz-Moreno E de la Paz Sánchez M 2013 The MADS transcription factor XAL2/AGL14 modulates auxin transport during Arabidopsis root development by regulating PIN expression. The EMBO Journal 32, 2884–2895. 24121311 10.1038/emboj.2013.216 PMC3817466

[CIT0022] Geldner N Friml J Stierhof YD Jurgens G Palme K 2001 Auxin transport inhibitors block PIN1 cycling and vesicle trafficking. Nature 413, 425–428. 11574889 10.1038/35096571

[CIT0023] Harrison BR Masson PH 2008 ARL2, ARG1 and PIN3 define a gravity signal transduction pathway in root statocytes. The Plant Journal 53, 380–392. 18047472 10.1111/j.1365-313X.2007.03351.x

[CIT0024] Hashiguchi Y Tasaka M Morita MT 2013 Mechanism of higher plant gravity sensing. American Journal of Botany 100, 91–100. 23115136 10.3732/ajb.1200315

[CIT0025] Higaki T Kutsuna N Sano T Kondo N Hasezawa S 2010 Quantification and cluster analysis of actin cytoskeletal structures in plant cells: role of actin bundling in stomatal movement during diurnal cycles in Arabidopsis guard cells. The Plant Journal 61, 156–165. 20092030 10.1111/j.1365-313x.2009.04032.x

[CIT0026] Hou G Kramer VL Wang YS Chen R Perbal G Gilroy S Blancaflor EB 2004 The promotion of gravitropism in *Arabidopsis* roots upon actin disruption is coupled with the extended alkalinization of the columella cytoplasm and a persistent lateral auxin gradient. The Plant Journal 39, 113–125. 15200646 10.1111/j.1365-313X.2004.02114.x

[CIT0027] Hou G Mohamalawari DR Blancaflor EB 2003 Enhanced gravitropism of roots with a disrupted cap actin cytoskeleton. Plant Physiology 131, 1360–1373. 12644685 10.1104/pp.014423 PMC166895

[CIT0028] Huang F Zago MK Abas L van Marion A Galván-Ampudia CS Offringa R 2010 Phosphorylation of conserved PIN motifs directs *Arabidopsis* PIN1 polarity and auxin transport. The Plant Cell 22, 1129–1142. 20407025 10.1105/tpc.109.072678 PMC2879764

[CIT0029] Jiang K Sorefan K Deeks MJ Bevan MW Hussey PJ Hetherington AM 2012 The ARP2/3 complex mediates guard cell actin reorganization and stomatal movement in *Arabidopsis* . The Plant Cell 24, 2031–2040. 22570440 10.1105/tpc.112.096263 PMC3442585

[CIT0030] Kiss JZ 2000 Mechanisms of the early phases of plant gravitropism. Critical Reviews in Plant Sciences 19, 551–573. 11806421 10.1080/07352680091139295

[CIT0031] Kiss JZ Hertel R Sack FD 1989 Amyloplasts are necessary for full gravitropic sensitivity in roots of *Arabidopsis thaliana* . Planta 177, 198–206. 11539759

[CIT0032] Kleine-Vehn J Ding Z Jones AR Tasaka M Morita MT Friml J 2010 Gravity-induced PIN transcytosis for polarization of auxin fluxes in gravity-sensing root cells. Proceedings of the National Academy of Sciences, USA 107, 22344–22349. 10.1073/pnas.1013145107 PMC3009804 21135243

[CIT0033] Kleine-Vehn J Leitner J Zwiewka M Sauer M Abas L Luschnig C Friml J 2008 Differential degradation of PIN2 auxin efflux carrier by retromer-dependent vacuolar targeting. Proceedings of the National Academy of Sciences, USA 105, 17812–17817. 10.1073/pnas.0808073105 PMC2584678 19004783

[CIT0034] Křeček P Skůpa P Libus J Naramoto S Tejos R Friml J Zažímalová E 2009 The PIN-FORMED (PIN) protein family of auxin transporters. Genome Biology 10, 249. 20053306 10.1186/gb-2009-10-12-249 PMC2812941

[CIT0035] Laxmi A Pan J Morsy M Chen R 2008 Light plays an essential role in intracellular distribution of auxin efflux carrier PIN2 in *Arabidopsis thaliana* . PLoS One 3, e1510. 18231596 10.1371/journal.pone.0001510 PMC2200863

[CIT0036] Le J El-Assal Sel D Basu D Saad ME Szymanski DB 2003 Requirements for *Arabidopsis* ATARP2 and ATARP3 during epidermal development. Current Biology 13, 1341–1347. 12906796 10.1016/s0960-9822(03)00493-7

[CIT0037] Le J Liu XG Yang KZ 2014 Auxin transport and activity regulate stomatal patterning and development. Nature Communications 5, 3090. 10.1038/ncomms4090 24463772

[CIT0038] Leitz G Kang BH Schoenwaelder ME Staehelin LA 2009 Statolith sedimentation kinetics and force transduction to the cortical endoplasmic reticulum in gravity-sensing *Arabidopsis* columella cells. The Plant Cell 21, 843–860. 19276442 10.1105/tpc.108.065052 PMC2671718

[CIT0039] Levine AJ Lubensky TC 2000 One- and two-particle microrheology. Physical Review Letters 85, 1774–1777. 10970611 10.1103/PhysRevLett.85.1774

[CIT0040] Li LJ Ren F Gao XQ Wei PC Wang XC 2013 The reorganization of actin filaments is required for vacuolar fusion of guard cells during stomatal opening in *Arabidopsis* . Plant Cell and Environment 36, 484–497. 10.1111/j.1365-3040.2012.02592.x 22891733

[CIT0041] Li X Li JH Wang W 2014 ARP2/3 complex-mediated actin dynamics is required for hydrogen peroxide-induced stomatal closure in Arabidopsis. Plant Cell and Environment 37, 1548–1560. 10.1111/pce.12259 24372484

[CIT0042] Lin D Nagawa S Chen J 2012 A ROP GTPase-dependent auxin signaling pathway regulates the subcellular distribution of PIN2 in *Arabidopsis* roots. Current Biology 22, 1319–1325. 22683260 10.1016/j.cub.2012.05.019 PMC3407329

[CIT0043] Mancuso S Barlow PW Volkmann D Baluska F 2006 Actin turnover-mediated gravity response in maize root apices: gravitropism of decapped roots implicates gravisensing outside of the root cap. Plant Signaling and Behavior 1, 52–58. 19521476 10.4161/psb.1.2.2432 PMC2633879

[CIT0044] Mathur J Mathur N Kirik V Kernebeck B Srinivas BP Hülskamp M 2003 Arabidopsis *CROOKED* encodes for the smallest subunit of the ARP2/3 complex and controls cell shape by region specific fine F-actin formation. Development 130, 3137–3146. 12783786 10.1242/dev.00549

[CIT0045] Mei Y Jia WJ Chu YJ Xue HW 2012 *Arabidopsis* phosphatidylinositol monophosphate 5-kinase 2 is involved in root gravitropism through regulation of polar auxin transport by affecting the cycling of PIN proteins. Cell Research 22, 581–597. 21894193 10.1038/cr.2011.150 PMC3292297

[CIT0046] Morita MT 2010 Directional gravity sensing in gravitropism. Annual Review of Plant Biology 61, 705–720. 10.1146/annurev.arplant.043008.092042 19152486

[CIT0047] Müller A Guan C Gälweiler L Tänzler P Huijser P Marchant A Parry G Bennett M Wisman E Palme K 1998 *AtPIN2* defines a locus of *Arabidopsis* for root gravitropism control. The EMBO Journal 17, 6903–6911. 9843496 10.1093/emboj/17.23.6903 PMC1171038

[CIT0048] Nakamura M Toyota M Tasaka M Morita MT 2011 An *Arabidopsis* E3 ligase, SHOOT GRAVITROPISM9, modulates the interaction between statoliths and F-actin in gravity sensing. The Plant Cell 23, 1830–1848. 21602290 10.1105/tpc.110.079442 PMC3123953

[CIT0049] Nebenführ A Ritzenthaler C Robinson DG 2002 Brefeldin A: deciphering an enigmatic inhibitor of secretion. Plant Physiology 130, 1102–1108. 12427977 10.1104/pp.011569 PMC1540261

[CIT0050] Ottenschläger I Wolff P Wolverton C Bhalerao RP Sandberg G Ishikawa H Evans M Palme K 2003 Gravity-regulated differential auxin transport from columella to lateral root cap cells. Proceedings of the National Academy of Sciences, USA 100, 2987–2991. 10.1073/pnas.0437936100 PMC151453 12594336

[CIT0051] Palmieri M Kiss JZ 2005 Disruption of the F-actin cytoskeleton limits statolith movement in *Arabidopsis* hypocotyls. Journal of Experimental Botany 56, 2539–2550. 16061504 10.1093/jxb/eri248

[CIT0052] Pollard TD 2007 Regulation of actin filament assembly by Arp2/3 complex and formins. Annual Review of Biophysics and Biomolecular Structure 36, 451–477. 10.1146/annurev.biophys.35.040405.101936 17477841

[CIT0053] Pollard TD Borisy GG 2003 Cellular motility driven by assembly and disassembly of actin filaments. Cell 112, 453–465. 12600310 10.1016/s0092-8674(03)00120-x

[CIT0054] Qiu J-L Jilk R Marks MD Szymanski DB 2002 The Arabidopsis *SPIKE1* gene is required for normal cell shape control and tissue development. The Plant Cell 14, 101–118. 11826302 10.1105/tpc.010346 PMC150554

[CIT0055] Rahman A Takahashi M Shibasaki K Wu S Inaba T Tsurumi S Baskin TI 2010 Gravitropism of *Arabidopsis thaliana* roots requires the polarization of PIN2 toward the root tip in meristematic cortical cells. The Plant Cell 22, 1762–1776. 20562236 10.1105/tpc.110.075317 PMC2910985

[CIT0056] Rakusová H Gallego-Bartolomé J Vanstraelen M Robert HS Alabadí D Blázquez MA Benková E Friml J 2011 Polarization of PIN3-dependent auxin transport for hypocotyl gravitropic response in *Arabidopsis thaliana* . The Plant Journal 67, 817–826 21569134 10.1111/j.1365-313X.2011.04636.x

[CIT0057] Reboulet JC Kumar P Kiss JZ 2010 *DIS1* and *DIS2* play a role in tropisms in *Arabidopsis thaliana* . Environmental and Experimental Botany 67, 474–478.

[CIT0058] Rigó G Ayaydin F Tietz O 2013 Inactivation of plasma membrane-localized CDPK-RELATED KINASE5 decelerates PIN2 exocytosis and root gravitropic response in *Arabidopsis* . The Plant Cell 25, 1592–1608. 23673979 10.1105/tpc.113.110452 PMC3694694

[CIT0059] Rotty JD Wu C Bear JE 2013 New insights into the regulation and cellular functions of the ARP2/3 complex. Nature Reviews Molecular Cell Biology 14, 7–12. 23212475 10.1038/nrm3492

[CIT0060] Sack FD 1997 Plastids and gravitropic sensing. Planta 203, S63–S68. 11540330 10.1007/pl00008116

[CIT0061] Sack FD Suyemoto MM Leopold AC 1986 Amyloplast sedimentation and organelle saltation in living corn columella cells. Americal Journal of Botany 73, 1692–1698. 11538871

[CIT0062] Saito C Morita MT Kato T Tasaka M 2005 Amyloplasts and vacuolar membrane dynamics in the living graviperceptive cell of the Arabidopsis inflorescence stem. The Plant Cell 17, 548–558. 15689424 10.1105/tpc.104.026138 PMC548825

[CIT0063] Sassi M Lu Y Zhang Y 2012 COP1 mediates the coordination of root and shoot growth by light through modulation of PIN1- and PIN2-dependent auxin transport in *Arabidopsis* . Development 139, 3402–3412. 22912415 10.1242/dev.078212

[CIT0064] Sato EM Hijazi H Bennett MJ Vissenberg K Swarup R 2015 New insights into root gravitropic signalling. Journal of Experimental Botany 66, 2155–2165. 25547917 10.1093/jxb/eru515 PMC4986716

[CIT0065] Sedbrook JC Chen R Masson PH 1999 *ARG1* (Altered Response to Gravity) encodes a DnaJ-like protein that potentially interacts with the cytoskeleton. Proceedings of the National Academy of Sciences, USA 96, 1140–1145. 10.1073/pnas.96.3.1140 PMC15364 9927707

[CIT0066] Shiva Kumar N Stevens MHH Kiss JZ 2008 Plastid movement in statocytes of the *arg1* (*altered response to gravity*) mutant. American Journal of Botany 95, 177–184. 21632343 10.3732/ajb.95.2.177

[CIT0067] Staiger CJ Blanchoin L 2006 Actin dynamics: old friends with new stories. Current Opinion in Plant Biology 9, 554–562. 17011229 10.1016/j.pbi.2006.09.013

[CIT0068] Sukumar P Edwards KS Rahman A Delong A Muday GK 2009 PINOID kinase regulates root gravitropism through modulation of PIN2-dependent basipetal auxin transport in Arabidopsis. Plant Physiology 150, 722–735. 19363095 10.1104/pp.108.131607 PMC2689958

[CIT0069] Swarup R Kramer EM Perry P Knox K Leyser HM Haseloff J Beemster GT Bhalerao R Bennett MJ 2005 Root gravitropism requires lateral root cap and epidermal cells for transport and response to a mobile auxin signal. Nature Cell Biology 7, 1057–1065. 16244669 10.1038/ncb1316

[CIT0070] Toyota M Ikeda N Sawai-Toyota S Kato T Gilroy S Tasaka M Morita MT 2013 Amyloplast displacement is necessary for gravisensing in Arabidopsis shoots as revealed by a centrifuge microscope. The Plant Journal 76, 648–660. 24004104 10.1111/tpj.12324

[CIT0071] Vanneste S Friml J 2009 Auxin: A trigger for change in plant development. Cell 136, 1005–1016. 19303845 10.1016/j.cell.2009.03.001

[CIT0072] Wan Y Jasik J Wang L Hao H Volkmann D Menzel D Mancuso S Baluška F Lin J 2012 The signal transducer NPH3 integrates the phototropin1 photosensor with PIN2-based polar auxin transport in Arabidopsis root phototropism. The Plant Cell 24, 551–565. 22374399 10.1105/tpc.111.094284 PMC3315232

[CIT0073] Wang HZ Yang KZ Zou JJ 2015 Transcriptional regulation of *PIN* genes by FOUR LIPS and MYB88 during *Arabidopsis* root gravitropism. Nature Communications 6, 8822. 10.1038/ncomms9822 PMC4673497 26578169

[CIT0074] Wirtz D 2009 Particle-tracking microrheology of living cells: principles and applications. Annual Review of Biophysics 38, 301–326. 10.1146/annurev.biophys.050708.133724 19416071

[CIT0075] Yamamoto K Kiss JZ 2002 Disruption of the actin cytoskeleton results in the promotion of gravitropism in inflorescence stems and hypocotyls of Arabidopsis. Plant Physiology 128, 669–681. 11842170 10.1104/pp.010804 PMC148928

[CIT0076] Yoder TL Zheng H-Q Todd P Staehelin LA 2001 Amyloplast sedimentation dynamics in maize columella cells support a new model for the gravity-sensing apparatus of roots. Plant Physiology 125, 1045–1060. 11161060 10.1104/pp.125.2.1045 PMC64904

[CIT0077] Zhang Z Friedman H Meir S Belausov E Philosoph-Hadas S 2011 Actomyosin mediates gravisensing and early transduction events in reoriented cut snapdragon spikes. Journal of Plant Physiology 168, 1176–1183. 21388706 10.1016/j.jplph.2011.01.019

[CIT0078] Zhao Y Pan Z Zhang Y 2013 The actin-related Protein2/3 complex regulates mitochondrial-associated calcium signaling during salt stress in *Arabidopsis* . The Plant Cell 25, 4544–4559. 24280386 10.1105/tpc.113.117887 PMC3875735

[CIT0079] Zheng Z Zou J Li H Xue S Wang Y Le J 2015 Microrheological insights into the dynamics of amyloplasts in root gravity-sensing cells. Molecular Plant 8, 660–663. 25704165 10.1016/j.molp.2014.12.021

[CIT0080] Zhu J Geisler M 2015 Keeping it all together: auxin–actin crosstalk in plant development. Journal of Experimental Botany 66, 4983–4998. 26085676 10.1093/jxb/erv308

