# Supplementary material for: The role of Arabidopsis Actin-Related Protein 3 in amyloplast sedimentation and polar auxin transport in root gravitropism
Source: J Exp Bot. 2016 Jul 29;67(18):5325–37. doi: 10.1093/jxb/erw294 (PMC5049384; doi:10.1093/jxb/erw294)
Supplement: Supplementary Data [file supp_67_18_5325__index.html]

The role of Arabidopsis Actin-Related Protein 3 in amyloplast sedimentation and polar auxin transport in root gravitropism — The role of Arabidopsis Actin-Related Protein 3 in amyloplast sedimentation and polar auxin transport in root gravitropism — Supplementary Data 

# The role of Arabidopsis Actin-Related Protein 3 in amyloplast sedimentation and polar auxin transport in root gravitropism

## Supplementary Data

Data files

- supplementary\_figures\_S1\_S6.pdf - Supplementary Data
