# Supplementary material for: The role of Arabidopsis Actin-Related Protein 3 in amyloplast sedimentation and polar auxin transport in root gravitropism
Source: J Exp Bot. 2016 Jul 29;67(18):5325–37. doi: 10.1093/jxb/erw294 (PMC5049384; doi:10.1093/jxb/erw294)
Supplement: Supplementary Data [file supp_erw294_supplementary_figures_S1_S6.pdf]

Supplemental Figure 1

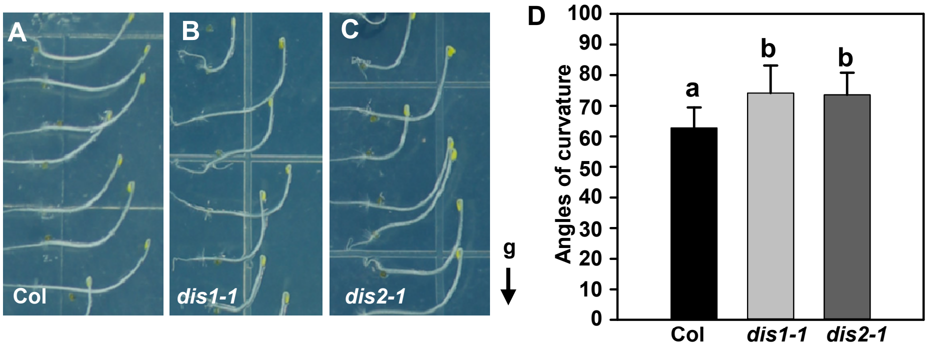

Supplemental Fig. 1 Hypocotyl gravitropic responses of Col, *dis1-1* and *dis2-1* plants.
(A-C) Hypocotyl gravitropic response of Col, *dis1-1*, *dis2-1* after 24 h of gravity stimulation. Three-day-old seedlings grown vertically in darkness were rotated 90º for 24 h to test their gravitropic responses. Arrow on the lower right indicates gravity direction.
(D) Angles of hypocotyl curvature after 90º reorientation for 24 h in darkness. Values are mean ± SD (*n* = 50–80 seedlings). Lowercase letters represent significantly different groups (one-way ANOVA test).

Supplemental Figure 2

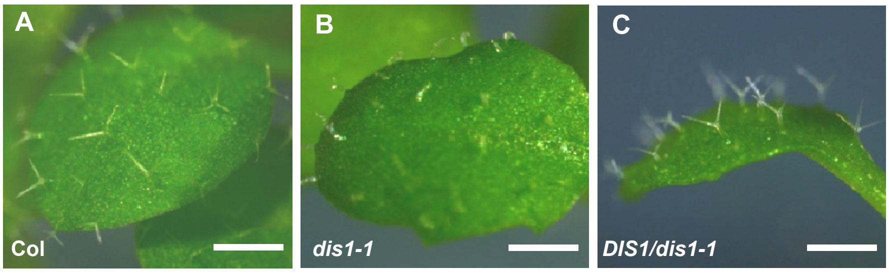

Supplemental Fig. 2 Phenotype complementation of the *dis1-1* mutant.
(A-C) Trichome phenotype of Col (**A**), *dis1-1* (**B**) and *dis1-1* complementation line (**C**). Scale bar = 0.5 mm.

Supplemental Figure 3

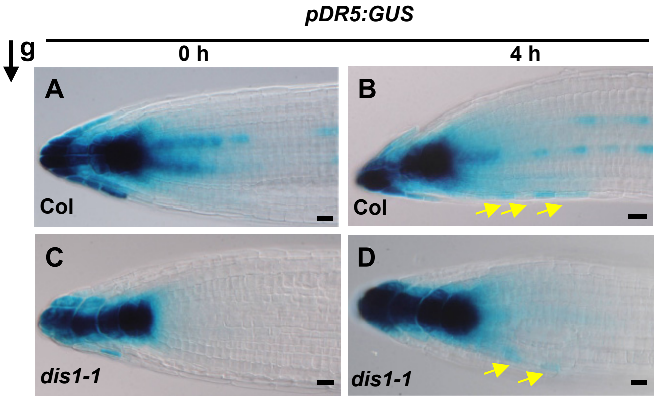

Supplemental Fig. 3 Asymmetric distribution of *pDR5::GUS* between the upper and lower sides of root tips after 90º reorientation.

(A-D) Representative images of *pDR5::GUS* expression in roots of Col and *dis1-1* after gravity stimulation for 4 h. Black arrow at left indicates the direction of gravity vector. The yellow arrows indicate the distribution of GUS expression on the lower side of root caps. Scale bar = 10 μm.

Supplemental Figure 4

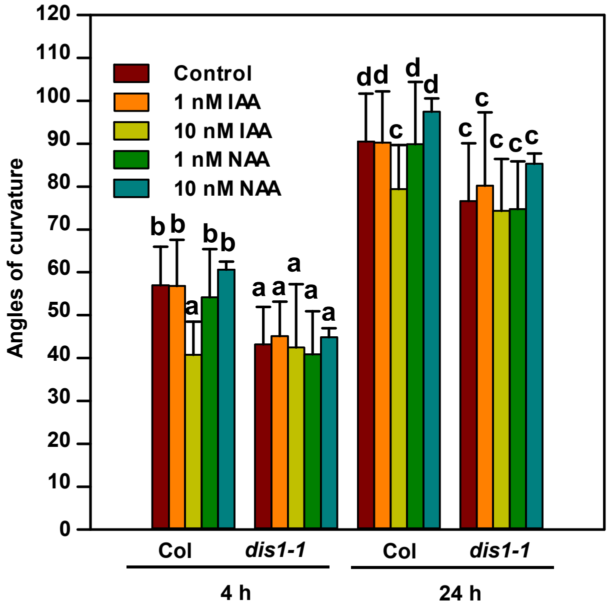

Supplemental Fig. 4 Exogenous IAA or NAA did not recover defect of root gravitropic response in *dis1-1* mutants. Three-day-old Col or *dis1-1* seedlings grown vertically on half-strength MS medium were transferred to 1/2 MS medium contain different concentration of IAA or NAA for 18 h, the seedlings were then reoriented for gravitropic response. The angles of root curvature were measured at indicated time points. Values are mean ± SD (*n* = 18–30 seedlings). Lowercase letters represent significantly different groups (one-way ANOVA test).

Supplemental Figure 5

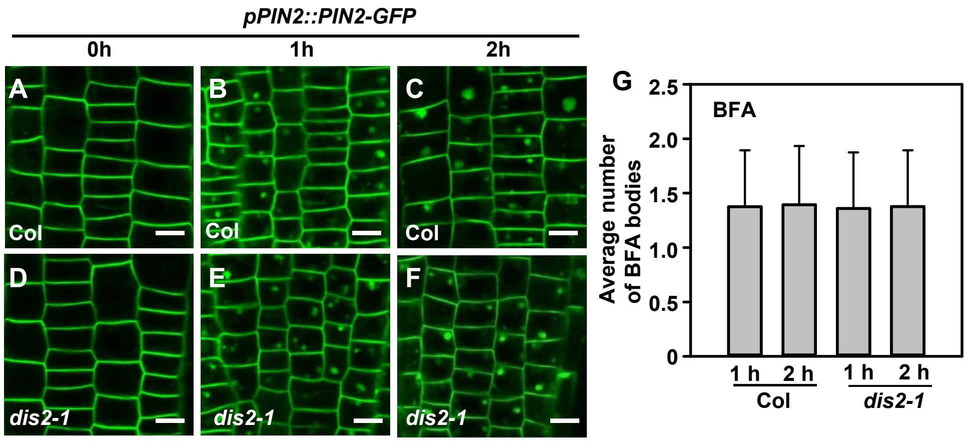

Supplemental Fig. 5 PIN2 cycling in *dis2-1* and wild-type plants.
(A-F) PIN2 cycling in the epidermal cells of *dis2-1* and wild-type plants. Internalization of PIN2 between Col (**A, B, C**) and *dis2-1* (**D, E, F**) after treatment with 50 μM BFA at indicated time points. Scale bar = 10 μm.
(G) Quantification of BFA bodies in Col and *dis2-1*. Values are mean ± D (*n* = 60–70 cells).

Supplemental Figure 6

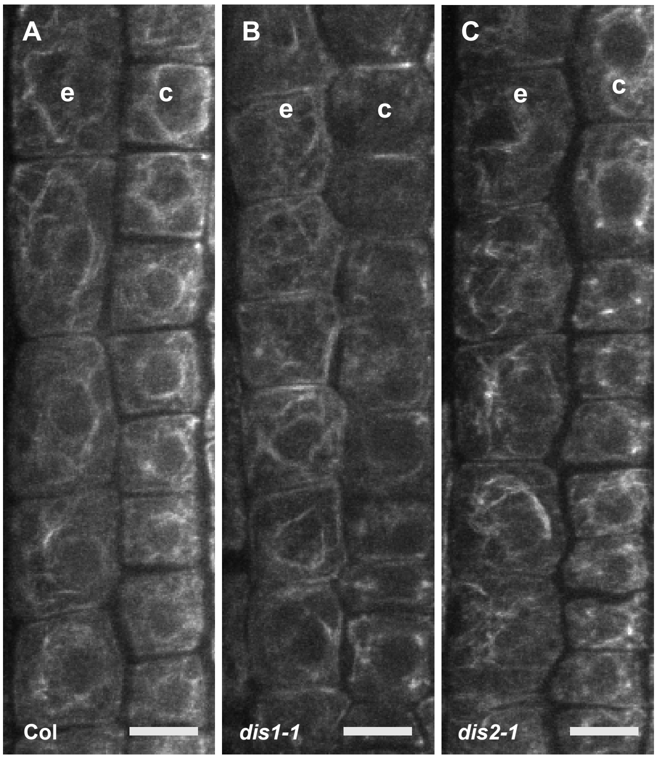

Supplemental Fig. 6 Actin cytoskeleton organization in the root transition zone of wild-type, *dis1-1* and *dis2-1.*

(A-C) Actin cytoskeleton organization in the root tip epidermis (e) and cortex (c) of Col **(A)**, *dis1-1* **(B)** and *dis2-1* **(C)**. Actin filaments in fixed root tips were labeled with Alexa Fluor-phalloidin dyes and viewed with a confocal microscope. Scale bar = 10 µm.
